# Biogeographic Role of the Kuroshio Current Intrusion in the Microzooplankton Community in the Boundary Zone of the Northern South China Sea

**DOI:** 10.3390/microorganisms9051104

**Published:** 2021-05-20

**Authors:** Ping Sun, Silu Zhang, Ying Wang, Bangqin Huang

**Affiliations:** 1 Key Laboratory of the Ministry of Education for Coastal and Wetland Ecosystem, College of the Environment and Ecology, Xiamen University, Xiamen 361102, China; zhangsilu@stu.xmu.edu.cn (S.Z.); wying@xmu.edu.cn (Y.W.); bqhuang@xmu.edu.cn (B.H.); 2 Fujian Province Key Laboratory for Coastal Ecology and Environmental Studies, College of the Environment and Ecology, Xiamen University, Xiamen 361102, China

**Keywords:** ciliate, protist, oligotrophic ocean, Kuroshio Current intrusion, South China Sea

## Abstract

Kuroshio Current intrusion (KCI) has significant impacts on the oceanographic conditions and ecological processes of the Pacific-Asian marginal seas. Little is known to which extent and how, specifically, the microzooplankton community can be influenced through the intrusion. Here, we focused on ciliates that often dominated the microzooplankton community and investigated their communities using high-throughput sequencing of 18S rRNA gene transcripts in the northern South China Sea (NSCS), where the Kuroshio Current (KC) intrudes frequently. We first applied an isopycnal mixing model to assess the fractional contribution of the KC to the NSCS. The ciliate community presented a provincial distribution pattern corresponding to more and less Kuroshio-influenced stations. Structural equation modeling revealed a significant impact of the KCI on the community, while environmental variables had a marginal impact. KCI-sensitive OTUs were taxonomically diverse but mainly belonged to classes *Spirotrichea* and *Phyllopharyngea*, suggesting the existence of core ciliates responding to the KCI. KCI-sensitive OTUs were grouped into two network modules that showed contrasting abundance behavior with the KC fraction gradient, reflecting differential niches (i.e., winner and loser) in the ciliate community during the Kuroshio intrusion scenarios. Our study showed that the Kuroshio intrusion, rather than environmental control, was particularly detrimental to the oligotrophic microzooplankton community.

## 1. Introduction

Oligotrophic oceans harbor one of the largest microbiomes on earth [1]. Understanding the mechanisms that control microbial communities in oligotrophic oceans is important [2]. In marine planktonic ecosystems, ciliates usually dominate the microzooplankton community, represent a morphologically fairly diverse assemblage of protists, and inhabit a great variety of marine environments [3]. They feed on bacteria and nanoflagellates, which in turn form the food of copepods and other small metazoans, serving as an essential link from the microbial loop to higher trophic levels [4].

The ciliate community in oligotrophic oceans reportedly exhibit a strong relationship with the environmental factors, e.g., temperature [5], salinity [6], nutrients [7], chlorophyll *a* [8], nanoflagellates [9], and bacteria [10]. Recent studies, however, have shown that these proximate factors are insufficient to explain the observed community distribution [11], which has led to attention being paid to the potential impacts of the movement of oceanic currents [12]. It has been reported that physical processes are particularly powerful, especially at a regional scale, and therefore have a strong influence on the distribution of microbial communities [13]. Different from zooplankton, microzooplankton, such as ciliates, are more influenced by the movement of ocean currents because of their limited swimming mobility [14]. Therefore, current intrusion into oligotrophic waters could exert a strong influence on the microzooplankton community through physical processes. Furthermore, besides vertical nutrients supply from the deep ocean, the lateral input of dissolved organic matter (DOM) by currents must be considered as an essential process for nutritional supplement in oligotrophic oceans. This lateral input of DOM by ocean currents has been reported to enhance particle export in the gyre of the eastern subtropical North Atlantic [15], increase the abundance and biomass of bacterioplankton in the Southern Brazilian Shelf [16], and enhance the abundance of phytoplankton and change the diatom community structure in the South Brazilian Bight [17]. Microzooplankton are different from readily responding microbes, e.g., bacteria and phytoplankton, and are generally not directly involved in the remineralization and utilization of DOMs but could still show a response through trophic cascading effects. To date, to which extent and how specifically the microzooplankton community in oligotrophic oceans can be influenced by lateral current intrusion remains poorly understood. Moreover, studies separating the effects of direct intrusion (i.e., physical processes) and indirect post-intrusion (i.e., biochemical conditions) on microzooplankton have not been explored.

The South China Sea (SCS) is the largest marginal sea of the western Pacific Ocean. The observed circulation patterns of the intermediate to upper layers of the SCS are primarily forced by the local monsoon systems and vary between seasons [18]. The Kuroshio Current (KC), the western boundary current of the North Pacific subtropical gyre, intrudes into the northern SCS (NSCS) when passing through the Luzon Strait, which is the deepest passage connecting the SCS with the Pacific Ocean [19]. Encountering the warm, nutrient-depleted, and DOM-rich KC, the NSCS provides an ideal experimental ground to explore the response of the microzooplankton community to the lateral intrusion into oligotrophic water. The Kuroshio Current intrusion (KCI) occurs all year round, although it varies in magnitude with stronger intrusion in winter than summer [19]. Previous studies have shown that the KCI influenced the nutrient biogeochemistry of the euphotic zone [20], induced enrichment of DOC [21], enhanced ammonia oxidation [22], influenced microbial activity (i.e., primary production, heterotrophic bacterial abundance, and bacterial production) [23], and changed the community structure of bacteria [24], picoplankton [25], and microphytoplankton [26] in the northern South China Sea. Microbes, e.g., bacteria and phytoplankton, can directly use resources from the KCI to support their growth and biogeochemical activities. For microzooplankton, the relationship between radiolarian assemblages and the KC has been relatively well documented in the marginal seas of the western Pacific [27,28,29]. The distribution of radiolarian assemblages was found to be strongly influenced by the KC in winter [30], while a released control in summer in the NSCS [31] and surface temperature, salinity, and annual silicate concentration were the most significant factors predicting the distribution of radiolarian communities [32]. However, much less is known about KCI impacts on the ciliate communities in NSCS. We speculated that, with the KCI, the input of DOM would be less important to ciliates than those microbes, e.g., bacteria and phytoplankton, which could directly use either DOM and/or products of the remineralization of the DOM input. Furthermore, given the background that the KC intrudes into the NSCS all year round, we hypothesized that the intrusion of the KC could facilitate high rates of dispersal of ciliates that inhabit it but strongly limit dispersal among currents, thereby dramatically changing the ciliate community composition along the intrusion route. Taken together, we hypothesized that the physical effect of the KCI is more important than its biochemical effect in governing ciliate biogeographic patterns. That is to say, intrusion, rather than post-intrusion, could be particularly detrimental for oligotrophic ciliate communities. However, evidence from field investigations of microzooplankton responses to lateral current intrusion is lacking. Here, we first applied an isopycnal mixing model to assess the fractional contribution of the KC to the NSCS. By employing high-throughput sequencing of rRNA gene transcripts, this study is the first to provide information on the spatial distribution of ciliate communities and the mechanism underlying lateral advection of ocean currents to modulate ciliate communities in the boundary zone of the NSCS.

## 2. Materials and Methods

### 2.1. Sampling and Biophysicochemical Analyses

**Figure 1:**
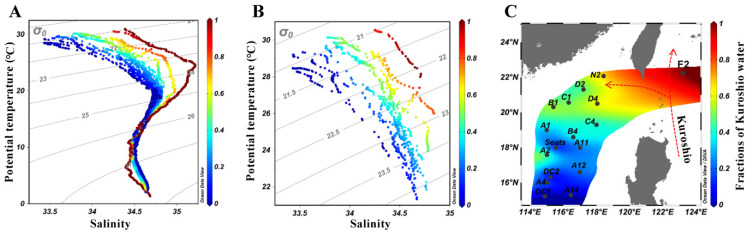
(**A**) The potential temperature versus salinity for the cruise stations in the summer of 2017. The lines in dark red and blue represent the two end-members, i.e., Kuroshio water and South China Sea water. (**B**) Distribution of potential temperature and salinity for sampling stations superimposed by Kuroshio fraction for waters above DCM layer. (**C**) The distribution pattern of average *R_K_* (Kuroshio fraction) above deep chlorophyll maximum for sampling stations.

The cruise was conducted onboard the R/V Dongfanghong II in the SCS from 11 July to 11 August 2017. In total, 16 stations were sampled in the NSCS (114.8 to 118.4° E, 15.2 to 22.0° N, Figure 1), which covered the intrusion route of Kuroshio water. We examined the chemical hydrography influenced by the Kuroshio on a spatial scale using hydrological parameters of temperature, salinity, and depth (Figure 1). Seawater samples were collected using a Seabird conductivity-temperature-depth (CTD) rosette system equipped with a 24 Niskin bottle sampler mounted with an oxygen sensor. Temperature, salinity, depth, and dissolved oxygen concentrations were measured using a CTD probe. At each station, 10 L of seawater was collected from the surface and the deep chlorophyll maximum (DCM), respectively (Table S1). The seawater was pre-filtered using a 200 µm nylon mesh (Sefar Nitex) to remove mesozooplankton and sequentially filtered through a 0.7 μm pore size polycarbonate filter (Millipore, Burlington, MA, USA). The filters were immediately shock frozen in liquid nitrogen and stored at −80 °C until further processing in the laboratory. Size and abundance analysis of nanoflagellates, including heterotrophic (HNFs) and pigmented (PNFs) nanoflagellates, followed Sherr et al. [33]. After pre-filtration with a 20 µm nylon mesh to exclude larger plankton, a 50 mL seawater was fixed with 1% final concentration of ice-cold glutaraldehyde and stained with DAPI (4′,6-diamidino-2-phenylindole; Sigma-Aldrich, St. Louis, MO, USA) for 10 min. The abundance and size of HNFs and PNFs were enumerated using fluorescence microscopy (Olympus Corporation, Tokyo, Japan). For bacterial abundance analysis, a 1.8 mL seawater was pre-filtered through a 20 µm mesh, mixed with ice-cold glutaraldehyde at a final concentration of 1% for 15 min in the dark, then stored at −80 °C, and later measured with flow cytometry (Beckman Coulter, Epics Altra II) with a 306C-5 argon laser (Coherent, Santa Clara, CA, USA). In the laboratory, samples were thawed at 37 °C and then stained with the SYBR Green I (1/10,000 final concentration) in the dark for 15 min at room temperature. Then, 10 µL fluorescent microspheres (diameter of 1 µm, 10^5^/mL, Molecular Probes, Eugene, OR, USA) were added to the 1 mL dyed samples as an internal standard. The samples were run at a flow rate of 0.1 to 1 mL h^−1^. The enumeration of bacterial abundance followed Marie et al. [34]. The discriminator was set to green fluorescence, and all parameters were set on logarithmic amplification. Heterotrophic bacteria were identified in the plots of red fluorescence vs. green fluorescence. Data analysis was performed using FlowJo vX.0.7 software (Tree Star, Ashland, OR, USA).

### 2.2. Assessment of the Kuroshio Fraction

To quantify the influence of the Kuroshio intrusion on the northern South China Sea, we adopted a well-validated, two-end-member isopycnal mixing model, which has been applied in many recent studies [22,23]. The model assumes that mixing between water masses is dominated by isopycnal mixing, whereas diapycnal mixing is relatively negligible [20]. According to Du et al. [20], two representative sites—F2 at 123.211° E, 22.243° N, and DC6 at 114.888° E, 15.233° N—were chosen to represent the Kuroshio and the SCS water, respectively (Figure 1). With this assumption, the fractional contributions of Kuroshio and SCS water could be derived by adopting the conservative along-isopycnal mixing law of *θ* or *S* (*θ* represents potential temperature, *S* represents salinity):

(1) $$R_{K}=\frac{\theta -\theta_{S}}{\theta_{K}-\theta_{S}}$$

(2) $$R_{K}=\frac{S-S_{S}}{S_{K}-S_{S}}$$

here, the Kuroshio water fraction is denoted as *R_K_*. *θ_K_* and *S_K_* are the endmember values of *θ* and *S* for the Kuroshio water, while *θ_S_* and *S_S_* represent those for the SCS proper water. Following Du et al. [20], *S* conservation was used for model prediction for the upper 100 m because the upper ocean potential temperature is affected by the seasonal heat flux [35].

### 2.3. RNA Extraction, PCR, and Sequencing

RNA was extracted from the filters using the AllPrep™ DNA/RNA Kit (Qiagen, Germantown, MD, USA) following the manufacturer’s instructions. The extracted RNA was treated with RQ1 RNase-Free DNase (Promega, Madison, WI, USA) to remove the remaining DNA and then reverse-transcribed into cDNA using a High-Capacity cDNA Reverse Transcription Kit (Applied Biosystems, Foster City, CA, USA). A nested PCR approach was employed to obtain the V4 region of the ciliate 18S rRNA gene transcript. First, the ciliate-specific primers 384F/1147R were used to amplify the ciliate partial 18S rRNA gene transcript [36]. Then, eukaryote universal primers were used to amplify the V4 region of the ciliate 18S rRNA gene transcript [37]. The PCR conditions used for the first and second PCRs follow Dopheide et al. [36] and Stoeck et al. [37], respectively. PCR amplicons were purified with a Wizard^®^ SV Gel and PCR Clean-Up System kit (Promega, Madison, WI, USA) and sent to MajorBio Bioinformatics Technology Co. Ltd. (Shanghai, China) for sequencing using the Illumina MiSeq platform. The original sequencing data are available at the NCBI Sequence Read Archive by accession code PRJNA719010.

### 2.4. Sequence Processing

Trimmomatic [38] and Flash [39] were used to clean the raw sequence data. Quality checking and filtering, demultiplexing, and assembly of data followed the following criteria: (i) low quality reads with an average quality score lower than 20 and that were shorter than 50 bp were removed; (ii) reads that contained ambiguous characters, a mismatch in barcode and/or two or more mismatches in primer, were discarded; (iii) reads with an overlapping region less than 10 bp, or with a mismatch ratio more than 0.2, were removed. Chimera and singleton removal and operational taxonomic unit (OTU) clustering were processed using USEARCH v10 [40]. Representative sequences were then clustered with a 97% sequence similarity, following Stoeck et al. [41]. Taxonomy assignments for the ciliate sequences were generated using SINTAX based on the Protist Ribosomal Reference database v4.7.1 (PR2) [42]. Non-ciliate OTUs were manually removed from the OTU table, and the generated ciliate-only OTU table was ready for further analysis. To normalize the sampling efforts, the OTU table was resampled to a minimum number of reads per sample, 7493 reads per sample, for statistical analyses. Phylogenetic trees were constructed according to the methods described by Logares et al. [43]. Briefly, OTU representative sequences were aligned with Mothur against an aligned template from SILVA [44]. TrimAl was then used to remove the poorly aligned sequences/nucleotides [45]. Phylogenetic trees were constructed using FastTree implemented in the QIIME [46].

### 2.5. Statistical Analyses

The sampling station map was plotted using the Ocean Data View (ODV) v4.7.8 [47]. All the following statistical analyses were carried out using R software v3.6.3. The pairwise geographic distance was computed using the “Imap” package with the longitude and latitude data of each sampling station [48]. Environmental factors were z-score normalized, and sequence data were transformed by log (x + 1) before downstream analyses. Partitioning of beta-diversity of ciliate communities was performed using principal coordinates analysis (PCoA) and an unweighted pair-group method with arithmetic means (UPGMA) cluster analysis using Bray–Curtis dissimilarities by the “vegan” package [49]. To determine the significance in the difference of community composition between two provincial groups, analysis of similarity (ANOSIM) was performed with the “vegan” package.

Structural equation modeling (SEM), a statistical technique to test hypothesized relationships among variables, was used to address our objective of exploring linkages between ciliate communities and physical–chemical–biological variables [50]. The model was constructed using the “lavaan” package [51]. We started with initial models that included all plausible pathways between ciliate community variation (represented by PCoA axes 1 and 2), relevant physical (*R_K_*), chemical (temperature, salinity, and DO), and biotic (bacterial abundance, size-fractionated HNF, and PNF abundance) factors. The fit of the model was tested using the maximum likelihood (χ^2^) goodness-of-fit test with *p*-values, Akaike information criteria (AIC), and the root mean square error of approximation (RMSEA).

To further confirm the relative importance of shaping factors on the total beta-diversity (i.e., the result inferred from SEM), three methods—partial Mantel tests, multiple regression on distance matrices (MRM), and variation partitioning analysis (VPA)—were performed. Partial Mantel tests and MRM were performed using the “ecodist” package, and VPA was performed using “vegan” [49,52]. The partial Mantel test estimates the correlation between two matrices controlling for the effects of a third matrix [53]. Thus, we can examine the pure spatial/environmental effects on the ciliate community by controlling the environmental/spatial matrix. The MRM approach was used to disentangle the separate effects of spatial and environmental variables on ciliate community composition. The similarity of spatial and individual environmental variables was regressed on the ciliate community similarity matrix to reveal the factors influencing community composition by permutations to determine the significance of the coefficients [54]. A canonical correspondence analysis (CCA)-based variation partitioning analysis (VPA) with an adjusted R^2^ coefficient was performed to assess the effect of spatial and environmental distances on community variation [55]. Principal coordinates of neighbor matrices (PCNM) analysis were used to extract a set of spatial variables. Variance inflation factor (VIF) analysis was used to remove factors with strong multicollinearity (VIF > 20). The importance of PCNM variables, environmental variables, and depth in explaining community variation was determined using a separate CCA analysis using Monte Carlo permutation tests with 9999 permutations. Only significant spatial and environmental variables were maintained for the VPA. In VPA, variation in the ciliate community was partitioned into pure effects of spatial and environmental variables and their interactions using unbiased estimators of the fractions [53].

Ecological processes (i.e., selection, dispersal, and drift) were quantified according to Stegen et al. [56]. To quantify the ecological processes shaping the ciliate community assembly, two steps were performed. First, the β-nearest taxon index (βNTI) for all pairwise community comparisons was calculated by quantifying the magnitude of deviation between the observed degree of phylogenetic turnover and the null distribution of phylogenetic turnover, determining the influence of selection. Second, Bray–Curtis-based Raup-Crick (RCbray) for pairwise community comparisons were evaluated by characterizing the magnitude of deviation between observed OTU composition turnover and null distribution of OTU composition turnover, assessing the influences of dispersal and drift [56].

To reveal the OTUs that characterized more and less Kuroshio-influenced stations, we used correlation-based “Indicator Species Analysis” with package “Indicspecies” [57]. Only OTUs with indicator values (IV) > 0.5, and *p* < 0.05, were considered good indicators. To obtain the strict differential OTUs, we also employed likelihood ratio tests (LRT) using the package “edgeR” in R [58]. OTUs whose abundances were identified as differing between more and less Kuroshio-influenced stations at a false discovery rate (FDR) corrected *p* < 0.05, were considered Kuroshio intrusion-responsive. The common OTUs identified by both methods were defined as Kuroshio-sensitive OTUs. We further explored the importance of KCI-sensitive OTUs, that is, their relative abundance in the whole community and their co-occurrence with other OTUs. OTUs were considered abundant when they comprised more than 1% of the reads in a sample and rare when they comprised less than 1% of the reads in all samples [59]. Only OTUs that occurred in at least 20% of samples and at least 32 reads (over the sample number) were included in the pairwise correlation analysis using the “Hmisc” package [60]. Robust correlations with Spearman’s correlation coefficients |r| > 0.6 and false discovery rate-corrected *p*-values < 0.01 were used to construct networks. Network visualization, modular analysis, and node-level topological properties were conducted using the interactive Gephi v0.9.2 [61].

## 3. Results

### 3.1. Background

The temperature and salinity at all sampling stations in the T-S diagram suggested a mixture of SCS and Kuroshio water, with all the sampling stations falling within the limits defined by the two representative end-members: SCS water (DC6, dark blue curve in Figure 1A) and Kuroshio water (F2, dark red curve in Figure 1A). This result indicates that the influence of Kuroshio Current (KC) should be properly examined using the two-end-member model (Figure 1B). The zonal distribution of *R_K_* in a contour plot showed an apparent spatial distribution pattern of the Kuroshio Current intrusion (KCI), with higher values to the east, indicating a clear pathway of the Kuroshio intrusion into the NSCS via the Luzon Strait (Figure 1C). Among the sampling stations, the estimated *R_K_* in the waters above DCM ranged from 0.01 at Station A14 to 0.68 at Station N2 (Figure 1C). Stations N2, D2, D4, C1, C4, B1, B4, A1, and A2, with *R_K_* values over 0.30, were classified as more KCI-influenced stations, while stations A11, A12, DC2, A4, A14, and DC6, with *R_K_* values less than 0.30, were classified as less KCI-influenced stations (Figure 1C). However, it should be noted that *R_K_* may vary by changing the two end-members. Here, the criterion of 0.30 was set as a boundary for more and less Kuroshio influence for convenient description and discussion.

### 3.2. Ciliate Biogeography

**Figure 2:**
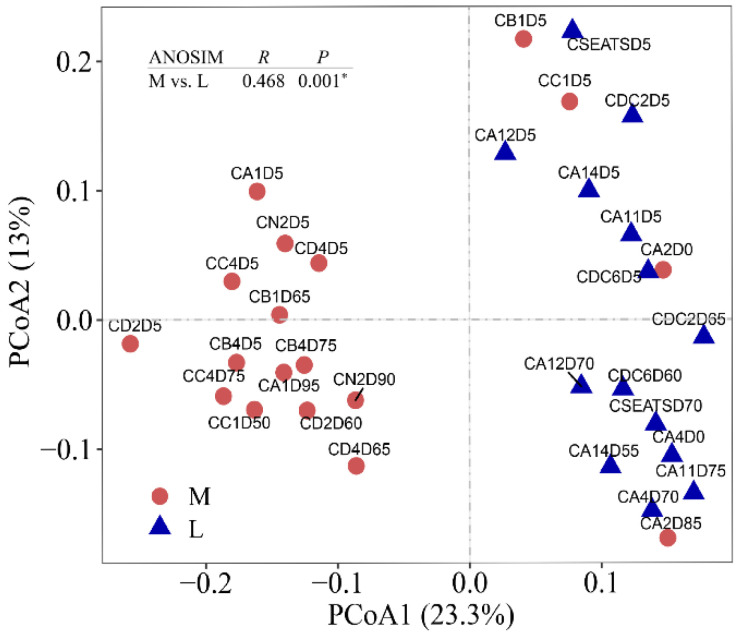
Bray–Curtis principal coordinate analysis (PCoA) plot illustrated the ciliate community distribution across the South China Sea, with analysis of similarity (ANOSIM) *R* and *p* values nested on the cage.

Altogether, the ciliate community profiling yielded 239,776 high-quality sequences, which belonged to 806 ciliate OTUs. We used PCoA and clustering analysis to visualize the differences between the ciliate communities (Figure 2 and Figure S1). Partitioning of community variation showed that ciliate communities fell into two regional clusters, that is, more KCI-influenced stations (corresponding to the stations in the northern region) and less KCI-influenced stations (corresponding to the stations in the southern region) (Figure 2). This division suggests that the sampling stations across the NSCS can be classified into two major ciliate provinces (Figure 2 and Figure S1). ANOSIM confirmed marked differences between the two ciliate provinces (ANOSIM: *R* = 0.468, *p* = 0.001). In conclusion, ciliates in the more and less KCI-influenced stations differed markedly in composition, exhibiting an apparent provincial distribution pattern.

### 3.3. Factors Shape Ciliate Biogeography

**Figure 3:**
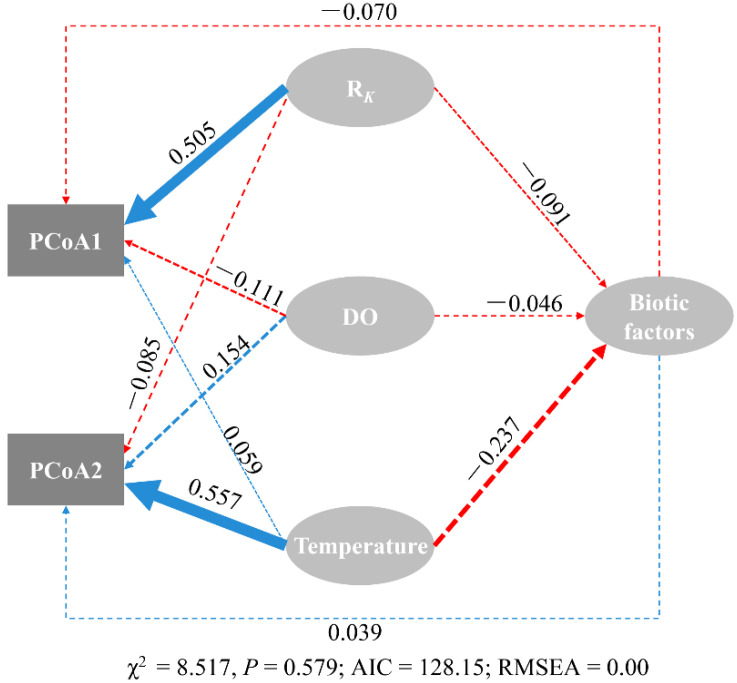
Directed graph of the structural equation modeling (SEM). Each oval shape represents an observed variable (i.e., measured) and latent variable (i.e., constructs). The loading for principal coordinate analysis (PCoA) scores of ciliate communities that create the latent variables are shown in the rectangle. Path coefficients are calculated after 1000 bootstraps and reflected in the solid width of the arrow, with blue and red indicating positive and negative effects, respectively. Dashed arrows show that coefficients did not differ significantly from 0 (*p* > 0.05). The model is assessed using the maximum likelihood (χ^2^) goodness-of-fit test with *p*-values, Akaike information criteria (AIC), and the root mean square error of approximation (RMSEA).

Structural equation modeling (SEM) analysis showed that the fraction of the KC and temperature had a significant direct effect on ciliate PCoA axes 1 and 2, respectively, with path coefficients of 0.505 and 0.557, respectively (Figure 3). Other factors showed no significant direct or indirect effects on either axis of the PCoA (Figure 3). The fit values of the model are shown in Figure 3. The results suggested a significant effect of the fraction of the KC on the ciliate community and a marginal effect of environmental variables (Figure 3). Three additional methods—partial Mantel tests, multiple regression on distance matrices (MRM), and variation partitioning analysis (VPA)—validated the robustness of the geographical effect (Table S2). This conclusion is consistent with the fact that the distribution pattern of the ciliate community is nonrandom on a regional scale (Figure 2). In other words, the two ciliate provinces reflected the fact that the dynamics of ciliates across the NSCS were governed primarily by spatial variability. The fact that the fraction of the KC strongly correlated with latitude (R^2^ = 0.804, *p* < 0.001) and the two ciliate provinces correspond to the fraction of the KC suggests that the apparent ciliate spatial distribution in the NSCS was the primary result of the Kuroshio intrusion (Table S2; Figure 1, Figure 2 and Figure S3). To conclude, ciliate biogeography was primarily driven by the physical processes of the KCI, whereas environmental variables had only a marginal effect.

### 3.4. KCI Sensitive OTUs

We further identified OTUs in the ciliate community whose abundances varied between more and less KCI-influenced stations. Indicator species analysis revealed high variability in the number of indicator OTUs between the two ciliate provinces, which were 95 and 59 in more and less KCI-influenced stations, respectively (Figure S3). As indicator OTUs were solely identified based on correlation, we validated them using likelihood ratio tests implemented in edgeR. EdgeR revealed 276 OTUs, which differed significantly in abundance between more and less KCI-influenced stations (Figure S3). Finally, we defined 151 OTUs supported by both methods as KCI-sensitive OTUs (Figure S3; Table S3). As an approximation of the effect size of the KCI on microbial communities, we calculated these sensitive OTUs to account for 4.9% of the total community sequences. KCI-sensitive OTUs comprised a high proportion of rare community members, with only 8.6% being abundant in the community. These KCI-sensitive organisms were taxonomically diverse but mainly belonged to the classes *Spirotrichea* and *Phyllopharyngea*, which accounted for 67% and 79% of sensitive OTUs in terms of numbers and relative abundance, respectively, indicating the existence of core ciliates responding to the KCI (Figure S4A,B). The taxonomic compositions of these strict indicators differed markedly between the two ciliate provinces, exhibiting a particular taxonomic pattern with the KCI (Figure S4C,D). Taken together, there were core ciliates responding to the KCI, and stations with more and less KCI influence support a specialized subset of ciliates. However, the majority of the ciliate community was shared between more and less KCI-influenced stations.

**Figure 4:**
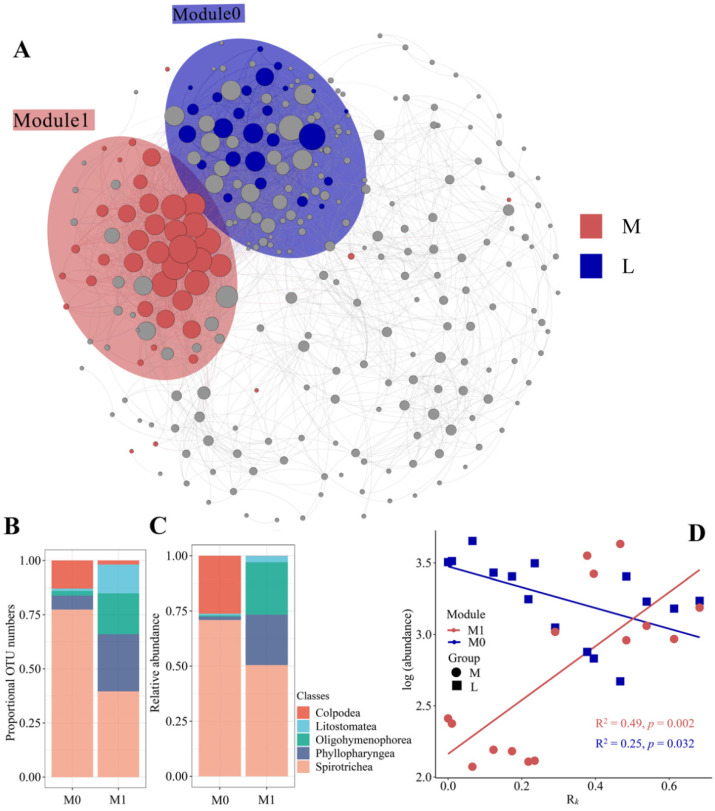
(**A**) The ciliate co-occurrence network. OTUs are colored by the KCI sensitive-OTUs types. Red and blue represent the sensitive OTUs in more and less Kuroshio-influenced stations, respectively. Shaded areas represent the network modules that KCI-sensitive OTUs accumulated. (**B**,**C**) Qualitative taxonomic composition of the KCI-sensitive modules is reported as proportional OTUs numbers (**B**) and relative abundance (**C**); (**D**) Regression relationship between *R_K_* and cumulative relative abundance of KCI-sensitive modules. M: more Kuroshio-influenced stations; L: less Kuroshio-influenced stations.

To further evaluate the importance of the KCI-sensitive OTUs, e.g., their co-occurrence with other species in the community, a correlation network was constructed. We partitioned the network into discrete modules and mapped the KCI-sensitive OTUs into the network. We observed that KCI-sensitive OTUs were grouped into two distinct modules—M1 and M0 (Figure 4A and Figure S5). M1 was enriched in more KCI-influenced stations, while M0 comprised sensitive OTUs specific to less KCI-influenced stations (Figure 4A and Figure S5). Although both modules comprised a taxonomically broad set of taxa, most OTUs belonged to the two classes, that is, *Spirotrichea* and *Phyllopharyngea*, indicating again that KCI targets specific ciliate lineages (Figure 4B,C). We explored the connectivity of KCI-sensitive OTUs in the network and their abundance in the community. The KCI-sensitive OTUs presented low to high degrees of co-occurrence in the network (Figure S6). Taken together, these results indicate that influential members of the ciliate community (i.e., members co-occurring with many others and members being abundant in the community) could be influenced by the Kuroshio intrusion. However, KCI-sensitive OTUs were mainly limited to rare community members despite the significant effect of KCI on community distribution and network pattern (Figure 2, Figure 3 and Figure 4; Table S2).

## 4. Discussion

### 4.1. Role of Intrusion and Post-Intrusion in Ciliate Biogeography

The ciliate community displayed a prominent provincial distribution across the NSCS, i.e., the northern and southern groups, strongly associated with the Kuroshio water fraction. Such a distribution pattern had been observed in the microphytoplankton community in this region [26]. This distribution pattern of ciliates along the Kuroshio fraction gradient suggests that the strong biogeographic provincialism of ciliates in the NSCS was shaped by the Kuroshio intrusion. The Kuroshio intrusion has been reported to change the bacterial composition [24] and picoplankton [25] communities in the NSCS. For microzooplankton, rhizarians, particularly polycystine radiolarians, which are the commonly seen members of the microzooplankton community, were closely associated with the pattern of water masses in the marginal seas of the western North Pacific [30,32,62]. In the present study, the SEM results indicated the primary role of the Kuroshio intrusion on the ciliate community (Figure 3). By employing three additional methods (i.e., partial Mantel test, MRM, and VPA), we confirmed the pronounced effect of spatial factors (i.e., geographic distance/latitude) on ciliate communities (Table S2). The effects of spatial factors could be attributed to the limited dispersal ability of microorganisms or the physical barriers that limit the dispersal of microorganisms [63]. Given the fact that the fraction of Kuroshio water is strongly associated with the latitude (Figure S2), this spatial effect, therefore, was primarily the result of the physical effect of the Kuroshio intrusion, that is, the movement of water masses, which act as dispersal barriers for ciliates [12,64]. The phylogenetic null model analysis supports this hypothesis, which showed that dispersal limitation was the most important ecological process and contributed to 55.0% of the community variance across the NSCS (Figure S7). Environmental variables such as temperature, salinity, DO, and bacterial abundance also explained the cluster pattern of the ciliate community (Table S2). Temperature and salinity are the main factors discriminating different water masses, again supporting the hypothesis that the movement of water masses might strongly shape ciliate biogeography in the NSCS. These results agree with those studies on radiolarians in the marginal seas of the northwestern Pacific [29,32]. Sea surface temperature and salinity were the most important factors affecting the composition of the radiolarian assemblages. The concentration of O_2_ and bacterial abundance are reported to be the factors tightly associated with heterotrophic activity [65,66]; therefore, they contribute to the formation of the spatial structure of the ciliate community. Taken together, compared with spatial factors, environmental control plays a much weaker role in shaping the structure of the ciliate community in the NSCS. In other words, the Kuroshio intrusion (physical effect) rather than post-intrusion (biochemical effect) was the most influential factor for the ciliate community across the NSCS.

It was noticed that there were four samples (i.e., surface samples of stations B1, C1, and A2; DCM sample of A2) that were exceptions to the provincial distribution pattern regarding the fraction of Kuroshio water (Figure 1, Figure 2 and Figure S1). Stations B1 and C1 were located on the shelf and slope regions of the NSCS. Previous studies have shown that a year-round northeastward current on the shelf region, known as the South China Sea Warm Current, has a strong influence on the upper current circulation in the NSCS, especially in the summertime [67]. A possible reason could be that the shallow water stations B1 and C1 might have been influenced by this northeastward current, exhibiting a community composition similar to that of the southern region. Station A2 was located on the edge of a warm eddy during the sampling period (Figure S8A). Warm and cold eddies are ubiquitous in the SCS [18]. Warm eddies, also called anticyclonic eddies, exhibit downward doming of isopycnal surfaces. These eddies contain warm, nutrient-depleted water with a lower phytoplankton abundance [68]. In contrast to the center, the edge of the warm eddy contains low temperature, high salinity, and nutrient-rich waters with higher phytoplankton abundance stimulated by upwelling at the eddy edges [69]. We examined the T-S profile and found an apparent upwelling at station A2, thus supporting our hypothesis (Figure S8B). Taken together, the relatively higher *R_K_* values of station A2 compared to the surrounding waters could be attributed to the edge effect of the warm eddy generating low temperature and high salinity characteristics of station A2. Therefore, the composition of station A2 was expected to be similar to that of less Kuroshio-influenced stations.

### 4.2. KCI-Sensitive Ciliates

A total of 151 OTUs were identified as KCI-sensitive OTUs in the ciliate community, which function as indicator taxa to explain the distribution pattern by the KCI (Figure S3). For example, a higher relative abundance of sensitive OTUs from the order Oligotrichida was found in more Kuroshio-influenced stations (Figure S4). Oligotrichid ciliates are important grazers of bacteria and nanoflagellates in the oceans [65,70]. Oligotrichid ciliates are approximately four times as abundant as loricated choreotrichid ciliates, and the relative abundance of the former tends to increase from coastal to open waters [71,72]. The Kuroshio Current is a narrow, strong westward boundary current of the North Pacific subtropical gyre. The open ocean waters it carried can be introduced into the NSCS during the intrusion. Therefore, the higher abundance of oligotrichid species may directly result from physical effects caused by the Kuroshio intrusion. Furthermore, the local environmental conditions would exert selection on these intruded species to determine whether they can be established in the new environment. The abundance of 3 to 10 μm HNF was higher in more Kuroshio-influenced stations than less Kuroshio-influenced stations (Figure S9) and showed a strong association with the relative abundance of sensitive-OTUs of Oligotrichida (r = −0.53, *p* = 0.02). These observations further indicate that these sensitive OTUs may benefit from the increased availability of resources and thus could be more abundant and could successfully inhabit the new environment to which they are introduced through water mass transport.

The relative abundance of sensitive OTUs from Choreotrichida was higher in the less KCI-influenced stations (Figure S4). Choreotrichida is phylogenetically divergent from Oligotrichida, and the species it harbors is common in both coastal and oceanic waters [71,73]. Choreotrichid ciliates are a significant consumer of algae and bacteria [74]. Some members of Choreotrichida are known to be voracious grazers and have been implicated in controlling the growth of prey populations, e.g., dinoflagellates [75] and diatoms [76] In the present study, the abundance of 10 to 20 μm pigmented nanoflagellates (PNF) was higher in less KCI-influenced stations compared to more KCI-influenced stations (Figure S9). The role of PNF grazers is consistent with the correlation between the relative abundance of sensitive choreotrichid OTUs and 10 to 20 μm PNF (r = −0.53, *p* = 0.04). In future studies, grazing experiments in the field will test the hypothesis regarding the functions of these sensitive OTUs.

To further evaluate the co-occurrence of sensitive OTUs, a correlation network was established. Two modules (i.e., M1 and M0) were identified to be enriched in more and less KCI-influenced stations, respectively (Figure 4). Their contrasting abundance behavior along the gradient of the fraction of the KC may represent differential niches in the ciliate community during the Kuroshio intrusion (Figure 4D). Previous studies have shown that modules comprise a subgroup of species with likely similar environmental requirements in positive co-occurrence networks [77]. The clustering of microbial species into distinct network modules has been used to infer physiochemical niches for various prokaryotic groups [78,79]. In the present study, the environmental variables showed marked differences between more and less KCI-influenced stations (Figure S9). The significant pairwise correlations within M1 and M0 were all positive (496 and 467 correlations in M1 and M0, respectively). In contrast, the correlations between the two modules were entirely negative (481 negative correlations), supporting the idea of the differential niches of the two modules. Taken together, we identified a subset of positive (M1) and negative (M0) associations between ciliate members and the Kuroshio intrusion, which might suggest that taxa within M1 could benefit from Kuroshio intrusion scenarios while that within M0 does the opposite, providing a list of “winner” and “loser” taxa.

## Data Availability

NCBI Sequence Read Archive, accession code PRJNA719010.

## Supplementary Materials

The following are available online at https://www.mdpi.com/article/10.3390/microorganisms9051104/s1, Figure S1: Hierarchical clustering analysis (UPGMA) revealed the provincial distribution pattern of the ciliate community. Figure S2: The linear regression analysis showed the relationship between latitude and RK Figure S3: The numbers of KCI sensitive OTUs were revealed by indicator species analysis (A) and EdgeR analysis (C), respectively. Venn diagram showed the common KCI sensitive OTUs revealed by both methods (B). Figure S4: The percentage of sensitive OTU numbers (A) and relative abundance of KCI sensitive OTUs (B) per class. Heat maps demonstrate the relative abundance of KCI sensitive OTUs at class level (C) and subclass or order level (D). Figure S5: Plots showing the number of OTUs in the top 7 most populated modules for the ciliate co-occurrence network. Figure S6: Frequency of degree of the KCI sensitive OTUs in ciliate co-occurrence network, Figure S7: Summary of the relative contributions of the ecological processes shaped the ciliate community assembly, Figure S8: Sea level anomaly (SLA, cm) in the South China Sea on 30 July 2017, Figure S9: Heat maps demonstrate the distribution of environmental variables between more and less Kuroshio-influenced stations. Table S1: The information of sampling stations in the northern South China Sea, Table S2. Partial Mantel test, multiple regression on dissimilarity matrices (MRM), and variation partitioning analysis (VPA) were conducted to reveal the relative importance of shaping factors on the total beta diversity of the ciliate community, Table S3. List of KCI sensitive OTUs identified by both indicator species analysis and EdgeR, R scripts for generating the Figure 2, Figure 3 and Figure 4.
