# Supplementary material for: Biogeographic Role of the Kuroshio Current Intrusion in the Microzooplankton Community in the Boundary Zone of the Northern South China Sea
Source: Microorganisms. 2021 May 20;9(5):1104. doi: 10.3390/microorganisms9051104 (PMC8161332; doi:10.3390/microorganisms9051104)
Supplement: Supplementary file 1 [file microorganisms-09-01104-s001.zip › microorganisms-1191065-supplementary.pdf]

Table S1. The information of sampling stations in the northern South China Sea.

Sampling station	Longitude(°E)	Latitude(°N)	Sampling layers (m)	Sampling date
A11	117.00	18.00	5	07/25/2017
	117.00	18.00	75	07/25/2017
A12	117.00	16.59	5	08/09/2017
	117.00	16.59	70	08/09/2017
A14	116.45	15.29	5	08/06/2017
	116.45	15.29	55	08/06/2017
A1	115.00	19.00	5	07/24/2017
	115.00	19.00	95	07/24/2017
A2	115.01	17.58	0	07/30/2017
	115.01	17.58	85	07/30/2017
A4	115.05	16.00	0	08/03/2017
	115.05	16.00	70	08/03/2017
B1	115.40	20.31	5	07/23/2017
	115.40	20.31	65	07/23/2017
B4	116.59	18.59	5	07/22/2017
	116.59	18.59	75	07/22/2017
C1	116.32	20.56	5	07/21/2017
	116.32	20.56	50	07/21/2017
C4	118.00	19.30	5	07/22/2017
	118.00	19.30	75	07/22/2017
D2	117.21	21.31	5	07/20/2017
	117.21	21.31	60	07/20/2017
D4	118.05	20.50	5	07/20/2017
	118.05	20.50	65	07/20/2017
DC2	115.24	16.31	5	08/02/2017
	115.24	16.31	65	08/02/2017
DC6	114.88	15.23	5	08/04/2017
	114.88	15.23	60	08/04/2017
N2	118.42	22.07	5	07/12/2017
	118.42	22.07	90	07/12/2017
Seats	115.56	18.00	5	07/25/2017
	115.56	18.00	70	07/25/2017

Table S2. Partial Mantel test, Multiple Regression on Dissimilarity Matrices (MRM), and Variation Partitioning Analysis (VPA) were conducted to reveal the relative importance of shaping factors on the total beta diversity of the ciliate community.

Partial Mantel test					MRM			VPA		
			Control for all other factors							
Variables	r	*P*	r	*P*	Variables	R^2	*P*	Variables	R^2	*P*
Geographic distance	**0.26**	**0.001**	**0.27**	**0**	Geographic distance	**7.05**	**0.001**	[G]	**0.189**	**0.001**
Environmental factors	0.056	0.202	**-**	**-**	Temperature	**6.65**	**0.002**	[E]	**0.147**	**0.001**
Depth	**0.109**	**0.038**	0.126	0.056	Dissolved oxygen	**2.07**	**0.008**	[D]	**0.057**	**0.001**
Temperature	**0.128**	**0.016**	0.073	0.293				[G|E+D]	**0.147**	**0.001**
Salinity	**0.095**	**0.037**	0.019	0.791				[E|G+D]	**0.093**	**0.001**
Dissolved oxygen	**0.319**	**0.001**	**0.308**	**0.001**				[D|G+E]	**0.031**	**0.001**
PNF_a	-0.03	0.626	**-**	**-**				Residuals	0.675	
PNF_{bc}	-0.12	0.961	**-**	**-**						
HNF_a	-0.06	0.755	**-**	**-**						
HNF_{bc}	-0.03	0.679	**-**	**-**						
Bacterial abundance	0.103	0.094	-	-						

PNF_a, HNF_a: the abundance of pigmented and heterotrophic nanoflagellates with a particle size of 2-5 μm.

PNF_{bc}, HNF_{bc}: the abundance of pigmented and heterotrophic nanoflagellates with a particle size of 5-20 μm.

[G]: geographic distance; [E]: environmental factors; [D]: depth; [G|E+D]: unique geographic distance; [E|G+D]: unique environmental factors; [D|G+E]: unique depth.

Table S3. List of KCI sensitive OTUs identified by both indicator species analysis and EdgeR.

OUT ID	Taxonomy	Group
Otu1003	Ciliophora-10; Ciliophora-10_X; Ciliophora-10_XX; Ciliophora-10_XXX; Ciliophora-10_XXX_sp.	More Kuroshio-influenced stations
Otu910	Colpodea; Colpodida; Colpodidae; Colpoda; Colpoda_sp.	More Kuroshio-influenced stations
Otu1406	Eukaryota; Alveolata; Ciliophora	More Kuroshio-influenced stations
Otu328	Eukaryota; Alveolata; Ciliophora	More Kuroshio-influenced stations
Otu905	Eukaryota; Alveolata; Ciliophora	More Kuroshio-influenced stations
Otu376	Litostomatea; Cyclotrichia	More Kuroshio-influenced stations
Otu495	Litostomatea; Cyclotrichia	More Kuroshio-influenced stations
Otu723	Litostomatea; Cyclotrichia	More Kuroshio-influenced stations
Otu855	Litostomatea; Cyclotrichia	More Kuroshio-influenced stations
Otu104	Litostomatea; Cyclotrichia; Cyclotrichia_X; Cyclotrichia_XX; Cyclotrichia_XX_sp.	More Kuroshio-influenced stations
Otu115	Litostomatea; Cyclotrichia; Cyclotrichia_X; Cyclotrichia_XX; Cyclotrichia_XX_sp.	More Kuroshio-influenced stations
Otu408	Litostomatea; Cyclotrichia; Cyclotrichia_X; Cyclotrichia_XX; Cyclotrichia_XX_sp.	More Kuroshio-influenced stations
Otu472	Litostomatea; Cyclotrichia; Cyclotrichia_X; Cyclotrichia_XX; Cyclotrichia_XX_sp.	More Kuroshio-influenced stations
Otu868	Litostomatea; Cyclotrichia; Cyclotrichia_X; Cyclotrichia_XX; Cyclotrichia_XX_sp.	More Kuroshio-influenced stations
Otu824	Litostomatea; Haptoria	More Kuroshio-influenced stations
Otu1012	Oligohymenophorea	More Kuroshio-influenced stations
Otu239	Oligohymenophorea	More Kuroshio-influenced stations
Otu831	Oligohymenophorea	More Kuroshio-influenced stations
Otu1344	Oligohymenophorea; Oligohymenophorea_X; Oligohymenophorea_XX	More Kuroshio-influenced stations
Otu813	Oligohymenophorea; Oligohymenophorea_X; Oligohymenophorea_XX	More Kuroshio-influenced stations
Otu70	Oligohymenophorea; Peritrichia; Zoothamniidae; Zoothamnium; Zoothamnium_pelagicum	More Kuroshio-influenced stations
Otu485	Oligohymenophorea; Scuticociliatia; Cinetochilidae; Cinetochilum	More Kuroshio-influenced stations
Otu1455	Oligohymenophorea; Scuticociliatia; Cyclidiidae; Cyclidium; Cyclidium_glaucoma	More Kuroshio-influenced stations
Otu305	Oligohymenophorea; Scuticociliatia; Orchitophryidae; Metanophrys; Metanophrys_sinensis	More Kuroshio-influenced stations

Otu840	Oligohymenophorea; Scuticociliatia; Scuticociliatia_X; Scuticociliatia_XX; Scuticociliatia_XX_sp.	More Kuroshio-influenced stations
Otu1041	Oligohymenophorea; Scuticociliatia; Scuticociliatia-1; Scuticociliatia-1_X; Scuticociliatia-1_X_sp.	More Kuroshio-influenced stations
Otu1086	Oligohymenophorea; Scuticociliatia; Scuticociliatia-1; Scuticociliatia-1_X; Scuticociliatia-1_X_sp.	More Kuroshio-influenced stations
Otu1313	Oligohymenophorea; Scuticociliatia; Scuticociliatia-1; Scuticociliatia-1_X; Scuticociliatia-1_X_sp.	More Kuroshio-influenced stations
Otu404	Oligohymenophorea; Scuticociliatia; Scuticociliatia-1; Scuticociliatia-1_X; Scuticociliatia-1_X_sp.	More Kuroshio-influenced stations
Otu433	Oligohymenophorea; Scuticociliatia; Scuticociliatia-1; Scuticociliatia-1_X; Scuticociliatia-1_X_sp.	More Kuroshio-influenced stations
Otu590	Oligohymenophorea; Scuticociliatia; Scuticociliatia-1; Scuticociliatia-1_X; Scuticociliatia-1_X_sp.	More Kuroshio-influenced stations
Otu624	Oligohymenophorea; Scuticociliatia; Scuticociliatia-1; Scuticociliatia-1_X; Scuticociliatia-1_X_sp.	More Kuroshio-influenced stations
Otu1329	Phyllopharyngea	More Kuroshio-influenced stations
Otu188	Phyllopharyngea	More Kuroshio-influenced stations
Otu267	Phyllopharyngea	More Kuroshio-influenced stations
Otu374	Phyllopharyngea	More Kuroshio-influenced stations
Otu912	Phyllopharyngea	More Kuroshio-influenced stations
Otu771	Phyllopharyngea; Cyrtophoria	More Kuroshio-influenced stations
Otu337	Phyllopharyngea; Cyrtophoria; Chlamydodontidae	More Kuroshio-influenced stations
Otu397	Phyllopharyngea; Cyrtophoria; Chlamydodontidae	More Kuroshio-influenced stations
Otu593	Phyllopharyngea; Cyrtophoria; Chlamydodontidae	More Kuroshio-influenced stations
Otu639	Phyllopharyngea; Cyrtophoria; Chlamydodontidae	More Kuroshio-influenced stations
Otu657	Phyllopharyngea; Cyrtophoria; Chlamydodontidae	More Kuroshio-influenced stations
Otu696	Phyllopharyngea; Cyrtophoria; Chlamydodontidae	More Kuroshio-influenced stations
Otu1617	Phyllopharyngea; Cyrtophoria; Chlamydodontidae; Chlamydodon; Chlamydodon_sp.	More Kuroshio-influenced stations
Otu292	Phyllopharyngea; Cyrtophoria; Chlamydodontidae; Chlamydodon; Chlamydodon_sp.	More Kuroshio-influenced stations
Otu400	Phyllopharyngea; Cyrtophoria; Chlamydodontidae; Chlamydodon; Chlamydodon_sp.	More Kuroshio-influenced stations
Otu542	Phyllopharyngea; Cyrtophoria; Chlamydodontidae; Chlamydodon; Chlamydodon_sp.	More Kuroshio-influenced stations
Otu548	Phyllopharyngea; Cyrtophoria; Chlamydodontidae; Chlamydodon; Chlamydodon_sp.	More Kuroshio-influenced stations
Otu717	Phyllopharyngea; Cyrtophoria; Chlamydodontidae; Chlamydodon; Chlamydodon_sp.	More Kuroshio-influenced stations

Otu745	Phyllopharyngea; Cyrtophoria; Chlamydodontidae; Chlamydodon; Chlamydodon_sp.	More Kuroshio-influenced stations
Otu153	Phyllopharyngea; Cyrtophoria; Chlamydodontidae; Chlamydodontidae_X; Chlamydodontidae_X_sp.	More Kuroshio-influenced stations
Otu707	Phyllopharyngea; Cyrtophoria; Hartmannulidae; Hartmannulidae_X; Hartmannulidae_X_sp.	More Kuroshio-influenced stations
Otu75	Phyllopharyngea; Suctoria	More Kuroshio-influenced stations
Otu279	Phyllopharyngea; Suctoria; Acinetidae; Acinetidae_X; Acinetidae_X_sp.	More Kuroshio-influenced stations
Otu799	Prostomatea; Prostomatea-1; Prostomatea-1_X; Prostomatea-1_XX; Prostomatea-1_XX_sp.	More Kuroshio-influenced stations
Otu1629	Spirotrichea	More Kuroshio-influenced stations
Otu310	Spirotrichea	More Kuroshio-influenced stations
Otu407	Spirotrichea	More Kuroshio-influenced stations
Otu422	Spirotrichea	More Kuroshio-influenced stations
Otu582	Spirotrichea	More Kuroshio-influenced stations
Otu841	Spirotrichea	More Kuroshio-influenced stations
Otu901	Spirotrichea	More Kuroshio-influenced stations
Otu661	Spirotrichea; Choreotrichida	More Kuroshio-influenced stations
Otu204	Spirotrichea; Choreotrichida; Choreotrichida_X; Choreotrichida_XX; Choreotrichida_XX_sp.	More Kuroshio-influenced stations
Otu904	Spirotrichea; Choreotrichida; Strobilidiidae_I	More Kuroshio-influenced stations
Otu247	Spirotrichea; Choreotrichida; Strobilidiidae_I; Pelagostrobilidium	More Kuroshio-influenced stations
Otu1510	Spirotrichea; Choreotrichida; Strombidinopsidae; Parastrombidinopsis; Parastrombidinopsis_minima	More Kuroshio-influenced stations
Otu270	Spirotrichea; Choreotrichida; Strombidinopsidae; Strombidinopsis	More Kuroshio-influenced stations
Otu311	Spirotrichea; Hypotrichia	More Kuroshio-influenced stations
Otu610	Spirotrichea; Hypotrichia	More Kuroshio-influenced stations
Otu1000	Spirotrichea; Strombidiida	More Kuroshio-influenced stations
Otu1331	Spirotrichea; Strombidiida	More Kuroshio-influenced stations
Otu1399	Spirotrichea; Strombidiida	More Kuroshio-influenced stations
Otu401	Spirotrichea; Strombidiida	More Kuroshio-influenced stations

Otu473	Spirotrichea; Strombidiida	More Kuroshio-influenced stations
Otu947	Spirotrichea; Strombidiida	More Kuroshio-influenced stations
Otu1152	Spirotrichea; Strombidiida; Strombidiidae; Strombidiidae_X; Strombidiidae_X_sp.	More Kuroshio-influenced stations
Otu85	Spirotrichea; Strombidiida; Strombidiidae_K; Strombidium_K; Strombidium_K_sp.	More Kuroshio-influenced stations
Otu97	Spirotrichea; Strombidiida; Strombidiidae_R; Strombidium_R; Strombidium_R_sp.	More Kuroshio-influenced stations
Otu117	Spirotrichea; Strombidiida; Tontoniidae_A; Spirotontonia; Spirotontonia_turbinata	More Kuroshio-influenced stations
Otu1181	Spirotrichea; Tintinnida	More Kuroshio-influenced stations
Otu1205	Spirotrichea; Tintinnida	More Kuroshio-influenced stations
Otu377	Spirotrichea; Tintinnida	More Kuroshio-influenced stations
Otu973	Spirotrichea; Tintinnida	More Kuroshio-influenced stations
Otu1105	Spirotrichea; Tintinnida; Eutintinnidae	More Kuroshio-influenced stations
Otu546	Spirotrichea; Tintinnida; Eutintinnidae; Eutintinnus; Eutintinnus_tubulosus	More Kuroshio-influenced stations
Otu1043	Spirotrichea; Tintinnida; TIN_01; Tintinnopsis_01; Tintinnopsis_dadayi	More Kuroshio-influenced stations
Otu1458	Spirotrichea; Tintinnida; TIN_02; Tintinnopsis_02; Tintinnopsis_radix	More Kuroshio-influenced stations
Otu988	Spirotrichea; Tintinnida; TIN_06; Tintinnopsis_06; Tintinnopsis_rara	More Kuroshio-influenced stations
Otu342	Spirotrichea; Tintinnida; Tintinnidiidae; Tintinnidium	More Kuroshio-influenced stations
Otu296	Spirotrichea; Tintinnida; Tintinnidiidae; Tintinnidium; Tintinnidium_sp.	More Kuroshio-influenced stations
Otu566	Spirotrichea; Tintinnida; Tintinnidiidae; Tintinnidium; Tintinnidium_sp.	More Kuroshio-influenced stations
Otu1268	Colpodea; Colpodea-1; Colpodea-1_X; Colpodea-1_XX; Colpodea-1_XX_sp.	Less Kuroshio-influenced stations
Otu1281	Colpodea; Colpodea-1; Colpodea-1_X; Colpodea-1_XX; Colpodea-1_XX_sp.	Less Kuroshio-influenced stations
Otu1376	Colpodea; Colpodea-1; Colpodea-1_X; Colpodea-1_XX; Colpodea-1_XX_sp.	Less Kuroshio-influenced stations
Otu446	Colpodea; Colpodea-1; Colpodea-1_X; Colpodea-1_XX; Colpodea-1_XX_sp.	Less Kuroshio-influenced stations
Otu477	Colpodea; Colpodea-1; Colpodea-1_X; Colpodea-1_XX; Colpodea-1_XX_sp.	Less Kuroshio-influenced stations
Otu534	Colpodea; Colpodea-1; Colpodea-1_X; Colpodea-1_XX; Colpodea-1_XX_sp.	Less Kuroshio-influenced stations
Otu538	Colpodea; Colpodea-1; Colpodea-1_X; Colpodea-1_XX; Colpodea-1_XX_sp.	Less Kuroshio-influenced stations
Otu629	Colpodea; Colpodea-1; Colpodea-1_X; Colpodea-1_XX; Colpodea-1_XX_sp.	Less Kuroshio-influenced stations

Otu708	Colpodea; Colpodea-1; Colpodea-1_X; Colpodea-1_XX; Colpodea-1_XX_sp.	Less Kuroshio-influenced stations
Otu750	Colpodea; Colpodea-1; Colpodea-1_X; Colpodea-1_XX; Colpodea-1_XX_sp.	Less Kuroshio-influenced stations
Otu964	Oligohymenophorea	Less Kuroshio-influenced stations
Otu681	Oligohymenophorea; Oligohymenophorea_X; Oligohymenophorea_XX	Less Kuroshio-influenced stations
Otu886	Oligohymenophorea; Oligohymenophorea_X; Oligohymenophorea_XX; Collinia; Collinia_sp.	Less Kuroshio-influenced stations
Otu381	Oligohymenophorea; Scuticociliatia	Less Kuroshio-influenced stations
Otu755	Oligohymenophorea; Scuticociliatia	Less Kuroshio-influenced stations
Otu747	Oligohymenophorea; Scuticociliatia; Scuticociliatia_X; Scuticociliatia_XX; Scuticociliatia_XX_sp.	Less Kuroshio-influenced stations
Otu118	Phyllopharyngea	Less Kuroshio-influenced stations
Otu1586	Phyllopharyngea	Less Kuroshio-influenced stations
Otu111	Phyllopharyngea; Cyrtophoria; Chilodonellidae; Pseudochilodonopsis; Pseudochilodonopsis_fluviatilis	Less Kuroshio-influenced stations
Otu503	Phyllopharyngea; Cyrtophoria; Chlamydodontidae	Less Kuroshio-influenced stations
Otu820	Phyllopharyngea; Cyrtophoria; Chlamydodontidae	Less Kuroshio-influenced stations
Otu326	Phyllopharyngea; Cyrtophoria; Chlamydodontidae; Chlamydodon; Chlamydodon_sp.	Less Kuroshio-influenced stations
Otu410	Phyllopharyngea; Cyrtophoria; Chlamydodontidae; Chlamydodon; Chlamydodon_sp.	Less Kuroshio-influenced stations
Otu1011	Spirotrichea	Less Kuroshio-influenced stations
Otu1223	Spirotrichea	Less Kuroshio-influenced stations
Otu163	Spirotrichea	Less Kuroshio-influenced stations
Otu320	Spirotrichea	Less Kuroshio-influenced stations
Otu484	Spirotrichea	Less Kuroshio-influenced stations
Otu521	Spirotrichea	Less Kuroshio-influenced stations
Otu643	Spirotrichea	Less Kuroshio-influenced stations
Otu669	Spirotrichea	Less Kuroshio-influenced stations
Otu737	Spirotrichea	Less Kuroshio-influenced stations
Otu862	Spirotrichea	Less Kuroshio-influenced stations

Otu1641	Spirotrichea; Choreotrichida	Less Kuroshio-influenced stations
Otu200	Spirotrichea; Choreotrichida	Less Kuroshio-influenced stations
Otu353	Spirotrichea; Choreotrichida	Less Kuroshio-influenced stations
Otu416	Spirotrichea; Choreotrichida	Less Kuroshio-influenced stations
Otu845	Spirotrichea; Choreotrichida	Less Kuroshio-influenced stations
Otu72	Spirotrichea; Choreotrichida; Leegaardiellidae_A; Leegaardiellidae_A_X; Leegaardiellidae_A_X_sp.	Less Kuroshio-influenced stations
Otu1076	Spirotrichea; Choreotrichida; Leegaardiellidae_B; Leegaardiella; Leegaardiella_sp.	Less Kuroshio-influenced stations
Otu1282	Spirotrichea; Choreotrichida; Leegaardiellidae_B; Leegaardiella; Leegaardiella_sp.	Less Kuroshio-influenced stations
Otu1518	Spirotrichea; Choreotrichida; Leegaardiellidae_B; Leegaardiella; Leegaardiella_sp.	Less Kuroshio-influenced stations
Otu156	Spirotrichea; Choreotrichida; Leegaardiellidae_B; Leegaardiella; Leegaardiella_sp.	Less Kuroshio-influenced stations
Otu243	Spirotrichea; Choreotrichida; Leegaardiellidae_B; Leegaardiella; Leegaardiella_sp.	Less Kuroshio-influenced stations
Otu402	Spirotrichea; Choreotrichida; Leegaardiellidae_B; Leegaardiella; Leegaardiella_sp.	Less Kuroshio-influenced stations
Otu448	Spirotrichea; Choreotrichida; Leegaardiellidae_B; Leegaardiella; Leegaardiella_sp.	Less Kuroshio-influenced stations
Otu476	Spirotrichea; Choreotrichida; Leegaardiellidae_B; Leegaardiella; Leegaardiella_sp.	Less Kuroshio-influenced stations
Otu491	Spirotrichea; Choreotrichida; Leegaardiellidae_B; Leegaardiella; Leegaardiella_sp.	Less Kuroshio-influenced stations
Otu990	Spirotrichea; Choreotrichida; Leegaardiellidae_B; Leegaardiella; Leegaardiella_sp.	Less Kuroshio-influenced stations
Otu210	Spirotrichea; Choreotrichida; Strobilidiidae_I	Less Kuroshio-influenced stations
Otu384	Spirotrichea; Choreotrichida; Strobilidiidae_I	Less Kuroshio-influenced stations
Otu452	Spirotrichea; Choreotrichida; Strobilidiidae_I	Less Kuroshio-influenced stations
Otu697	Spirotrichea; Choreotrichida; Strobilidiidae_I	Less Kuroshio-influenced stations
Otu1099	Spirotrichea; Choreotrichida; Strobilidiidae_I; Pelagostrobilidium; Pelagostrobilidium_sp.	Less Kuroshio-influenced stations
Otu712	Spirotrichea; Choreotrichida; Strombidinopsidae	Less Kuroshio-influenced stations
Otu1097	Spirotrichea; Euplotia; Uronychiidae; Uronychia	Less Kuroshio-influenced stations
Otu552	Spirotrichea; Hypotrichia	Less Kuroshio-influenced stations
Otu896	Spirotrichea; Hypotrichia	Less Kuroshio-influenced stations

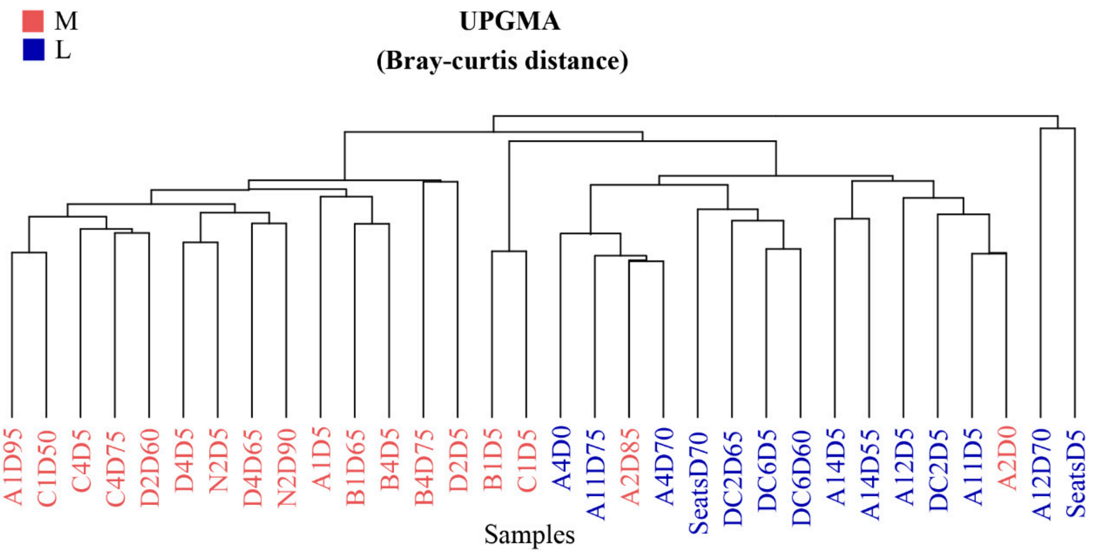

Figure S1. Hierarchical clustering analysis (UPGMA) revealed the provincial distribution pattern of the ciliate community. M: more Kuroshio-influenced stations; L: less Kuroshio-influenced stations.

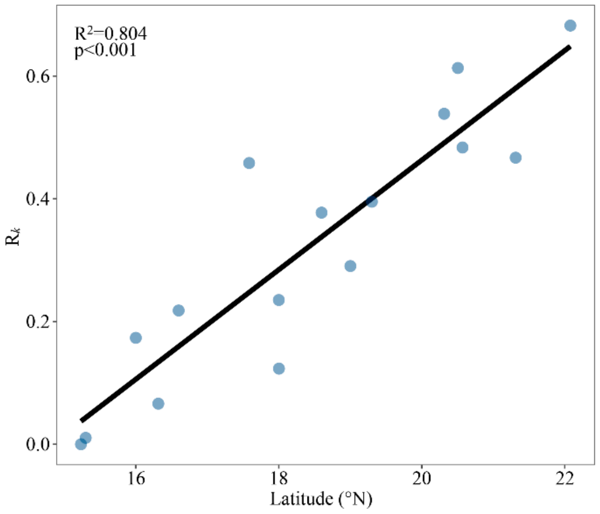

Figure S2. The linear regression analysis showed the relationship between latitude and R_k.

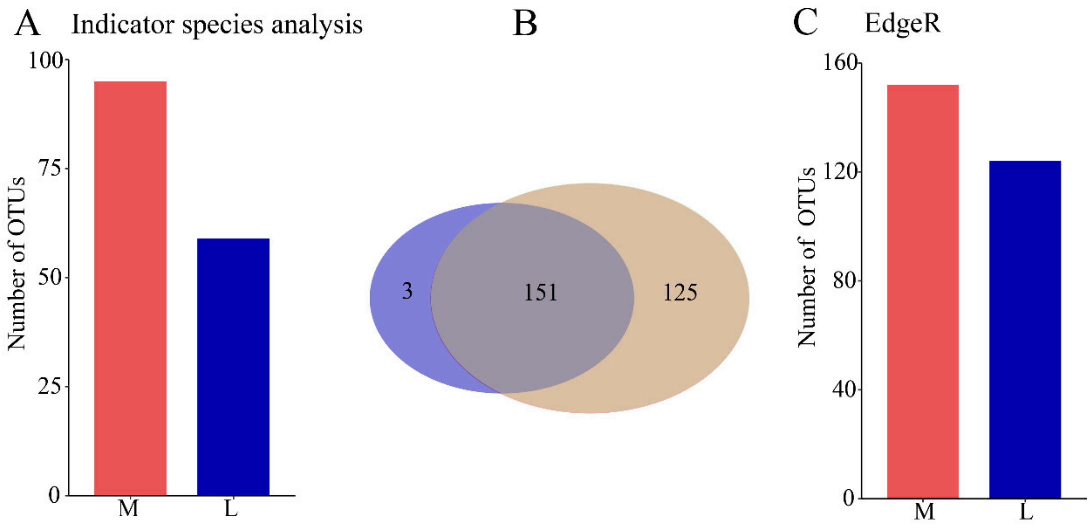

Figure S3. The numbers of KCI sensitive OTUs were revealed by indicator species analysis (A) and EdgeR analysis (C), respectively. Venn diagram showed the common KCI sensitive OTUs revealed by both methods (B). M: more Kuroshio-influenced stations; L: less Kuroshio-influenced stations.

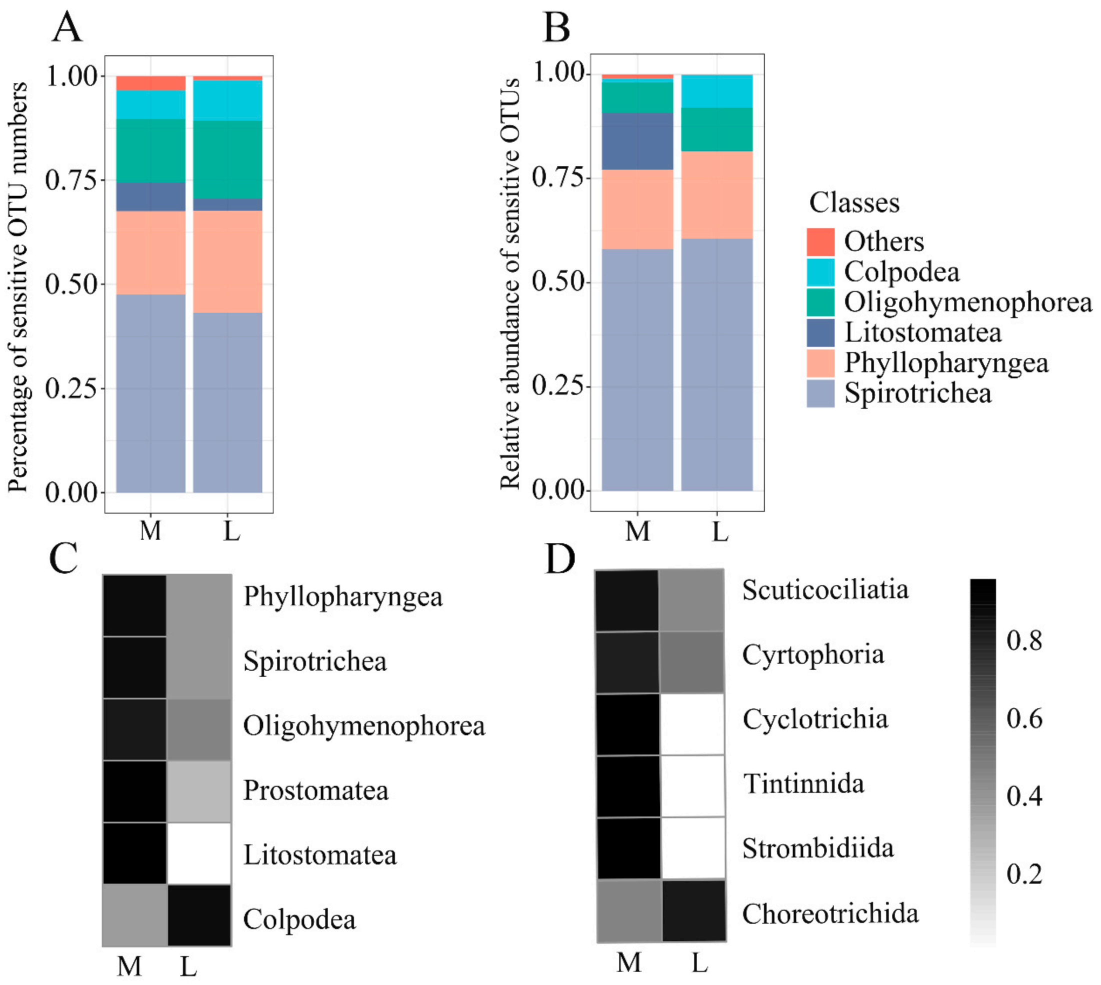

Figure S4. The percentage of sensitive OTU numbers (A) and relative abundance of KCI sensitive OTUs (B) per class. Heat maps demonstrate the relative abundance of KCI sensitive OTUs at class level (C) and subclass or order level (D). M: more Kuroshio-influenced stations; L: less Kuroshio-influenced stations.

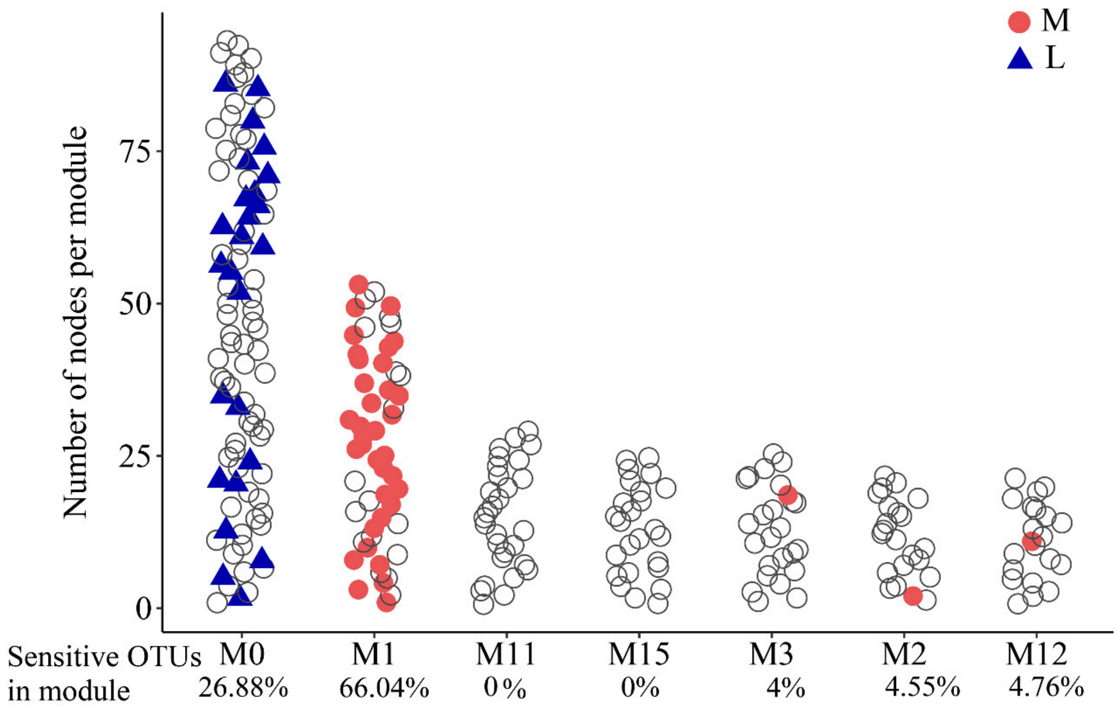

Figure S5. Plots showing the number of OTUs in the top 7 most populated modules for the ciliate co-occurrence network. Red circles and blue triangles represent sensitive OTUs in more and less Kuroshio-influenced stations, respectively.

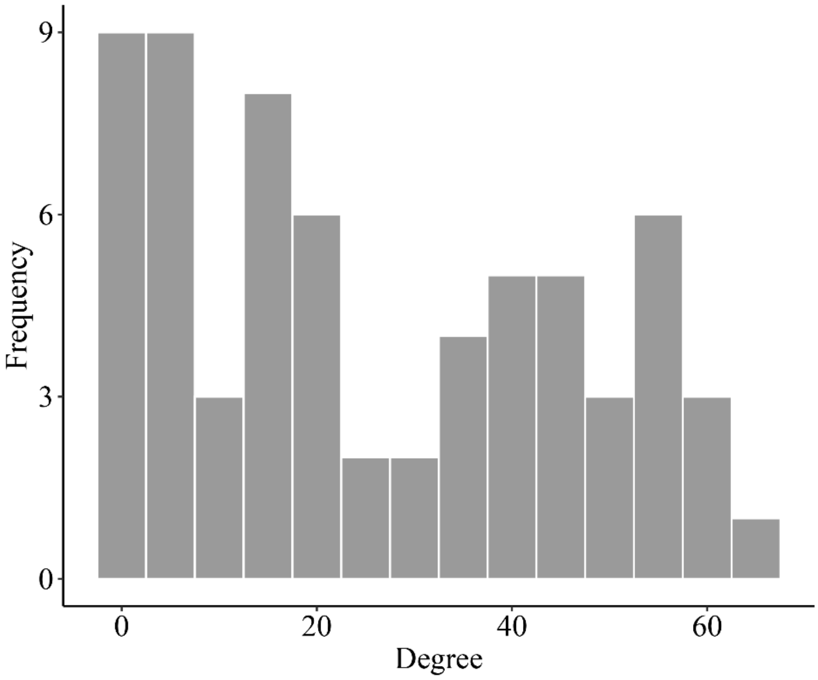

Figure S6. Frequency of degree of the KCI sensitive OTUs in ciliate co-occurrence network.

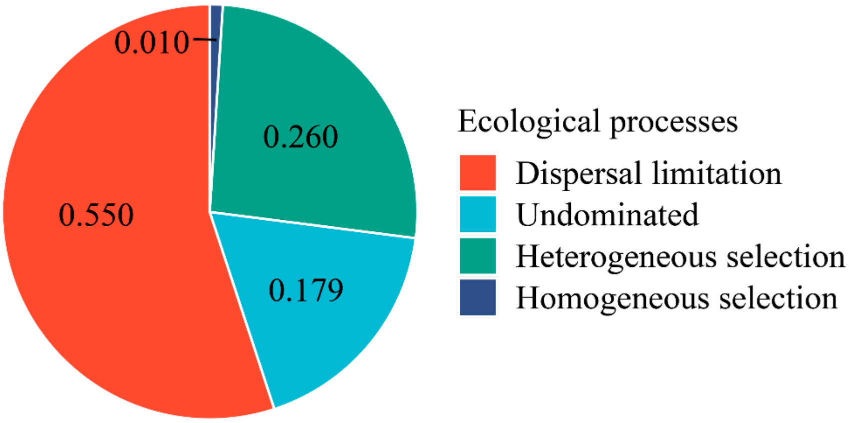

Figure S7. Summary of the relative contributions of the ecological processes shaped the ciliate community assembly.

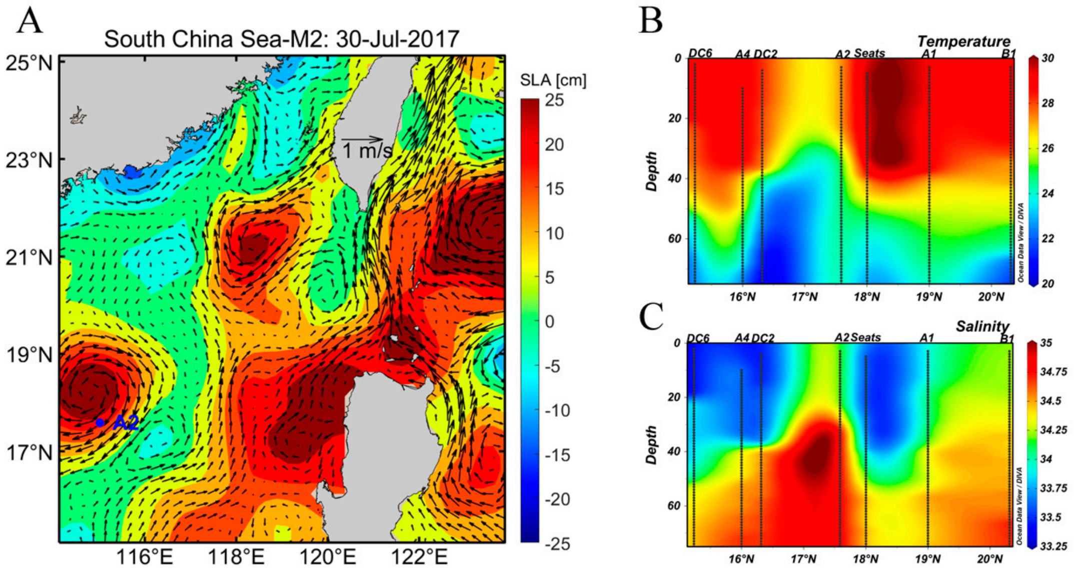

Figure S8. Sea level anomaly (SLA, cm) in the South China Sea on July 30th, 2017 (A). Vertical distribution of temperature (B) and salinity (C) at station A2 and nearby stations.

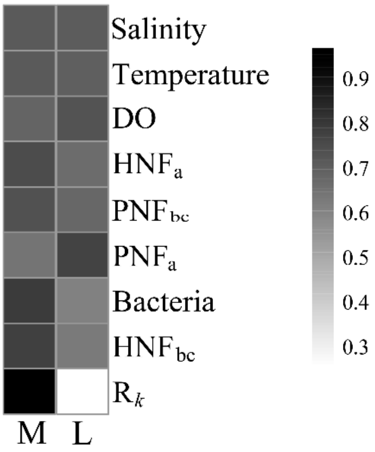

Figure S9. Heat maps demonstrate the distribution of environmental variables between more and less Kuroshio-influenced stations. M: more Kuroshio-influenced stations; L: less Kuroshio-influenced stations.

R scripts for generating figures 2-4 in the manuscript.

```
# Plot figure 2:
# Load the packages
library(ape)
library(ggplot2)
library(ggrepel)
library(vegan)
library(maptools)
# Import data and grouping file
otu <- read.table(file = "out.table.txt", header = T, row.names = 1, sep = "\t")
otu <- log(otu + 1)
group <- read.delim("group.txt", header = T, row.names = 1)
group$Group <- factor(group$Group, levels = c("M", "L"))
# Calculate bray-curtis distance of community
otu.dist <- vegdist(t(otu), scale = T, method = "bray", na.rm = T)
# Principal coordinates analysis
PCOA <- pcoa(otu.dist, correction = "none", rn = NULL)
result <- PCOA$values[, "Relative_eig"]
pro1 <- as.numeric(sprintf("%.3f", result[1])) * 100
pro2 <- as.numeric(sprintf("%.3f", result[2])) * 100
x <- PCOA$vectors
sample_names <- rownames(x)
pc <- as.data.frame(PCOA$vectors)
pc$names <- sample_names
legend_title <- ""
pc$Axis.1 <- pc$Axis.1
pc$Axis.2 <- pc$Axis.2
pc$Group <- group$Group
xlab <- paste("PCoA1 (", pro1, "%)", sep = "")
```

```
ylab <- paste("PCoA2 (", pro2, "%)", sep = "")
# Plot
p <- ggplot(pc, aes(-Axis.1, Axis.2)) +
  geom_point(aes(color = Group, shape = Group), size = 4) +
  geom_text_repel(aes(label = names), size = 3, vjust = -1) +
  labs(x = xlab, y = ylab, title = "PCoA", color = legend_title) +
  geom_hline(yintercept = 0, linetype = 4, color = "grey") +
  geom_vline(xintercept = 0, linetype = 4, color = "grey") +
  theme_bw() + ## Set font size and color
  theme(axis.text.x = element_text(size = 16, family = "serif", color = "black")) +
  theme(axis.text.y = element_text(size = 16, family = "serif", color = "black")) +
  theme(axis.title.x = element_text(size = 16, family = "serif", color = "black")) +
  theme(axis.title.y = element_text(size = 16, family = "serif", color = "black")) +
  theme(legend.text = element_text(size = 14, family = "serif", color = "black")) +
  theme(legend.title = element_text(size = 14, hjust = 0, family = "serif", color =
"black")) +
  theme(strip.text = element_text(size = 16, family = "serif", color = "black"))
p <- p + scale_color_manual(values = c("#CD5555", "#0000AA")) # Set point color
p

# Plot figure 3:
# Data preparation
# Environmental factors (except Rk) were log (x + 1) transformed, and then were
#combined with the PCoA1 and PCoA 2 axes to generate a new data named
#"log.pc.csv".
# Structural equation modeling analysis
library(lavaan)
DATA <- read.csv("log.pc.csv", row.names = 1, header = T)
# Fit Confirmatory Factor Analysis Models (CFA)
# Assumed latent variable
```

```
cfa_model <- "
bio = ~ PNFa + PNFbc + HNFa + HNFbc + bacteria
"
# Execute CFA
cfa_fit <- cfa(model = cfa_model, data = DATA)
summary(cfa_fit)
# Check the results and remove the insignificant factors.
cfa_model <- "
bio = ~ PNFa + PNFbc + HNFa
"
# Execute CFA
cfa_fit <- cfa(model = cfa_model, data = DATA)
summary(cfa_fit)
# Assumed variable structure
sem_model <- "
#Latent variables
bio = ~ PNFa + PNFbc + HNFa
#Regressions
bio ~ Rk + temperature + DO
PC1 ~ Rk + temperature + DO + bio
PC2 ~ Rk + temperature + DO + bio
"
# Execute SEM
sem_fit <- sem(
  model = sem_model, data = DATA, se = "bootstrap", bootstrap =
    1000
)
summary(sem_fit, standardized = TRUE, fit.measures = TRUE, rsquare = TRUE)
# Model fit
fitMeasures(sem_fit, c("chisq", "pvalue", "aic", "rmsea"))
```

Plot the graph with the Adobe Illustrator.

```
# Plot figure 4A:
# Only OTUs that occur in at least 20% of samples and at least have 32 reads (over the number of samples) are included in the network analysis.
library(Hmisc)
Abu <- read.table("network-otu.txt", header = T, row.names = 1, sep = "\t")
# Load function
source("Pairwise_correlations.R")
# Calculate OTU correlations
pattern <- co_occurrence_network(Abu, 0.6, 0.01)
write.graph(pattern$graph3, "Pos0.6-total.gml", format = "gml")
# Plot co-occurrence network with Gephi.

# Plot figure 4B and 4C
# Qualitative taxonomic composition of the KCI sensitive modules is reported as
# proportional OTUs numbers and relative abundance.
# Load packages
library(ggplot2)
library(ggsci)
# Import data
data <- read.delim("module 0 and 1.txt", header = T)
names(data)
data$class <- factor(data$class, levels = rev(unique(data$class)))
# Plot figure 4B:
p1 <- ggplot(data = data, aes(x = Modularity, y = The.number.of.OTUs, fill = class), width = 0.5) + geom_col(position = "fill")
p1 <- p1 + theme_bw() + labs(x = "", y = "Proportional OTUs number")
p1 <- p1 + theme(axis.text.x = element_text(size = 16, family = "serif", colour = "black")) +
```

```
    theme(axis.text.y = element_text(size = 16, family = "serif", colour = "black")) +
    theme(axis.title.x = element_text(size = 16, family = "serif", colour = "black")) +
    theme(axis.title.y = element_text(size = 16, family = "serif", colour = "black")) +
    theme(legend.text = element_text(size = 15, family = "serif", colour = "black")) +
    theme(legend.title = element_text(size = 14, hjust = 0, family = "serif", colour = "black")) +
    theme(strip.text = element_text(size = 16, family = "serif", colour = "black"))
p1 <- p1 + scale_fill_npg(alpha = 0.8)
p1

# Plot figure 4C:
p2 <- ggplot(data = data, aes(x = Modularity, y = The.relative.abudance, fill = class), width =0.5) + geom_col(position = "fill")
p2 <- p2 + theme_bw() + labs(x = "", y = "Proportional relative abundance")
p2 <- p2 + theme(axis.text.x = element_text(size = 16, family = "serif", colour = "black")) +
    theme(axis.text.y = element_text(size = 16, family = "serif", colour = "black")) +
    theme(axis.title.x = element_text(size = 16, family = "serif", colour = "black")) +
    theme(axis.title.y = element_text(size = 16, family = "serif", colour = "black")) +
    theme(legend.text = element_text(size = 15, family = "serif", colour = "black")) +
    theme(legend.title = element_text(size = 14, hjust = 0, family = "serif", colour = "black")) +
    theme(strip.text = element_text(size = 16, family = "serif", colour = "black"))
p2 <- p2 + scale_fill_npg(alpha = 0.8)
p2

# Plot figure 4D (remove the outlier A2).
library(ggplot2)
library(vegan)
# Import data
```

```
data <- read.delim("otu.module 0 and 1.mean remove A2.txt", header = T)
data$Group <- factor(data$Group, levels = c("M", "L"))
# plot scatter diagram
p1 <- ggplot(data, aes(x = Rk, y = log.abundance, color = Module)) +
  geom_smooth(method = "lm", formula = y ~ x, se = F) +
  scale_linetype_manual() +
  geom_point(aes(size = 4, shape = Group)) +
  scale_shape_manual(values = c(16, 15)) +
  theme_classic() +
  labs(x = "Rk", y = "log (abundance)")
# Set font size and color
p1 <- p1 + theme(axis.text.x = element_text(size = 16, family = "serif", color = "black"))
+
  theme(axis.text.y = element_text(size = 16, family = "serif", color = "black")) +
  theme(axis.title.x = element_text(size = 16, family = "serif", color = "black")) +
  theme(axis.title.y = element_text(size = 16, family = "serif", color = "black")) +
  theme(legend.text = element_text(size = 14, family = "serif", color = "black")) +
  theme(legend.title = element_text(size = 14, hjust = 0, family = "serif", color =
"black")) +
  theme(strip.text = element_text(size = 16, family = "serif", color = "black"))
p1 <- p1 + scale_color_manual(values = c("#0000AA", "#CD5555")) # Set points
color
p1
# Calculate the regression coefficient of each module
M0 <- subset(data, Module == "M0")
M1 <- subset(data, Module == "M1")
fm1 <- lm(log.abundance ~ Rk, data = M0)
summary(fm1)
fm2 <- lm(log.abundance ~ Rk, data = M1)
summary(fm2)
```

The statistic values were added to the figure using the Adobe Illustrator.
